# Intraocular pressure elevation precedes a phagocytosis decline in a model of pigmentary glaucoma

**DOI:** 10.12688/f1000research.13797.2

**Published:** 2018-04-09

**Authors:** Yalong Dang, Susannah Waxman, Chao Wang, Priyal Shah, Ralitsa T. Loewen, Nils A. Loewen

**Affiliations:** 1 Department of Ophthalmology, University of Pittsburgh School of Medicine, Pittsburgh, PA, 15213, USA; 2 Department of Ophthalmology, Xiangya Hospital of Central South University, Changsha, Hunan, 410008, China

**Keywords:** Pigment dispersion glaucoma, aqueous outflow, trabecular meshwork, intraocular pressure, phagocytosis

## Abstract

**Background: **Outflow regulation and phagocytosis are key functions of the trabecular meshwork (TM), but it is not clear how the two are related in secondary open angle glaucomas characterized by an increased particle load. We hypothesized that diminished TM phagocytosis is not the primary cause of early ocular hypertension and recreated pigment dispersion in a porcine *ex vivo* model.

**Methods: **Sixteen porcine anterior chamber cultures received a continuous infusion of pigment granules (Pg), while 16 additional anterior chambers served as controls (C). Pressure transducers recorded the intraocular pressure (IOP). The phagocytic capacity of the trabecular meshwork was determined by fluorescent microspheres.

**Results:** The baseline IOPs in Pg and C were similar ( *P*=0.82). A significant IOP elevation occurred in Pg at 48, 120, and 180 hours (all *P*<0.01, compared to baseline). The pigment did not cause a reduction in TM phagocytosis at 48 hours when the earliest IOP elevation occurred, but at 120 hours onward ( *P*=0.001 compared to C). This reduction did not result in an additional IOP increase at 120 or 180 hours compared to the first IOP elevation at 48 hours ( *P*>0.05).

**Conclusions: **In this porcine model of pigmentary glaucoma, an IOP elevation occurs much earlier than when phagocytosis fails, suggesting that two separate mechanisms might be at work.

## Introduction

The conventional outflow is guarded by the trabecular meshwork (TM), a complex three dimensional, layered tissue that contains variable amounts of extracellular matrix (ECM) ^1^. The aqueous passes from the anterior chamber into Schlemm's canal (SC) by entering first the uveal TM (UTM), the corneoscleral TM (CTM) and finally the juxtacanalicular TM (JCT) ^2^. The JCT contains proteoglycans and hyaluronans and presents the aqueous humor with an increasingly tighter fluid passageway towards the SC in a process referred to as funneling ^3^. The aqueous eventually passes the inner wall of SC endothelium mainly by two different mechanisms, a paracellular route in between endothelial cells and a transcellular route ^4^ consisting of intracellular pores and giant vacuoles that are time and pressure dependent ^1, 5, 6^. Failure of the TM and the inner wall of SC endothelial cells to maintain homeostasis and a normal cytoskeleton and can cause ocular hypertension ^7^. For instance, pigment dispersion ^8^ and corticosteroids can increase and contract actin stress fibers and result in an elevation of the intraocular pressure (IOP) ^8, 9^. Conversely, relaxing the cytoskeleton with Rho kinase inhibitors can reverse these effects ^10, 11^. Phagocytosis of debris is another function of TM cells ^1^. A chronic phagocytosis demand in the form of pigment ^12^, erythrocyte-derived ghost cells ^13^, inflammatory cells ^14^, photoreceptor outer segments ^15^, lens and pseudoexfoliation material ^16, 17^ can all lead to secondary glaucomas even though the amount of material itself is unlikely to cause a physical outflow obstruction. Although these glaucomas make for a sizable fraction of open angle glaucomas, it was difficult to study the cellular mechanism that leads to an IOP elevation.

We recently developed a porcine *ex vivo* pigmentary glaucoma (PG) model that recreates the IOP elevation, stress fiber formation, and phagocytosis reduction that are characteristic of human PG ^8^. A gene expression analysis indicated an activation of the RhoA signaling pathway and a downstream effect of tight junction formation negatively regulated by RhoA-mediated actin cytoskeletal reorganization ^8^. In the current study, we hypothesized that early ocular hypertension from pigment dispersion is the result of actin cytoskeletal changes and occurs before phagocytosis declines.

## Methods

### Pig eye perfusion culture and pigmentary glaucoma model

This study was conducted in accordance with the Association for Research in Vision and Ophthalmology Statement for the Use of Animals in Ophthalmic and Vision Research. Because no live vertebrate animals were used and pig eyes were acquired from a local abattoir (Thoma Meat Market, Saxonburg, PA), no Institutional Animal Care and Use approval was required.

Thirty-two porcine eyes were cultured within 2 hours of enucleation. Extraocular tissues were removed, and the eyes were decontaminated with 5% povidone-iodine solution (CAT# 3955-16, United States Pharmacopeia, Rockville, MD) for two minutes and washed three times in phosphate buffered saline (PBS). Posterior segments, lenses, and irises were removed and the anterior segments with intact TM mounted in the perfusion system as previously described ^8, 18, 19^. We used the same method to generate pigment granules as recently described in a model of pigmentary glaucoma (PG) ^8^. Briefly, pigment granules were produced by subjecting the iris to freeze-thaw and resuspension washing before dilution of the stock to a final concentration of 1.67×10 ^7^ particles/ml. Eyes in the pigment dispersion group were continuously perfused with pigment added to the culture medium for up to 180 hours (Pg) and compared to controls (C). The perfusate consisted of Dulbecco's modified Eagle media (DMEM, SH30284, HyClone, GE Healthcare, UK) supplemented with 1% FBS and 1% antibiotics (15240062, Thermo Fisher Scientific, Waltham, MA) at a constant rate of 3 µl/min using a microinfusion pump (PHD 22/2000; Harvard Apparatus, Holliston, MA). IOP was measured intracamerally by a pressure transducer (SP844; MEMSCAP, Skoppum, Norway) and recorded at two-minute intervals (LabChart, ADInstruments, Colorado Springs, CO). Baseline IOPs were obtained after IOP stabilization for 48 hours.

### TM phagocytosis

The *in situ* TM phagocytosis was measured using an epifluorescence microscope after microsphere perfusion. In brief, a suspension of 0.5 μm carboxylate-modified yellow-green fluorescent microspheres ^20 ^(CAT# F8813, Thermo Fisher, Waltham, MA) at 5×10 ^8^ particles/ml was added to the perfusate at 48, 120, and 180 hours and perfused for 24 hours. The eyes were removed from their perfusion dishes, washed three times with pre-warmed PBS, secured again in the perfusion dishes, and placed upside down for imaging. The TM, visualized from the underside of the transparent perfusion dish, was photographed and measured by acquiring the images with a camera and epifluorescence equipped dissecting fluorescence microscope (SZX16, Olympus, Tokyo, Japan) at a 680×510 pixel resolution and a 200 ms exposure. The mean fluorescence intensity was quantified by ImageJ (Version 1.50i, NIH) as previously described ^21^ at 48, 120, and 180 hours by measuring the fluorescence intensity in the TM.

To validate that the microspheres were phagocytosed by TM cells, the TM was dissected and digested with collagenase type IA (C9891, Sigma Aldrich, St. Louis, MO) at 2mg/ml and 1% FBS for 30 min at room temperature. The cells were filtered with a 70-micron cell strainer and resuspended in 0.5 ml of PBS. The percentage of TM cells that had ingested fluorescent microspheres was determined using flow cytometry.

To get a more accurate visualization of the phagocytosed microbeads, we used confocal microscopy. TM cells were seeded into the wells of a six-well plate and fixed with 4% PFA. The cell membranes were labeled with *Lycopersicon esculentum* agglutinin (TL; Texas red-conjugated; #TL-1176, Vector Laboratories, Inc., Burlingame, CA) at room temperature for 1 hour. The cell nuclei were counterstained with DAPI (D1306, Thermo Fisher Scientific, Waltham, MA). Photos and 3D videos were taken using an upright laser scanning confocal at 400x magnification (BX61, Olympus, Tokyo, Japan).

### Histology

After the TM phagocytosis assay, the anterior segments were fixed with 4% PFA for 24 hours, washed three times with PBS, dehydrated in 70% ethanol, and embedded in paraffin. Sections were cut to a thickness of 5 μm and stained with hematoxylin and eosin (H&E).

### Statistics

Data were presented as the mean ± standard error and analyzed by PASW Statistics 18 (SPSS Inc., Chicago, IL). The baseline IOP was compared to the other time points of the same eye using a paired *t*-test. Other quantitative data were analyzed by one-way ANOVA. A *p* value ≤ 0.05 was considered statistically significant.

## Results

**Figure 1.**
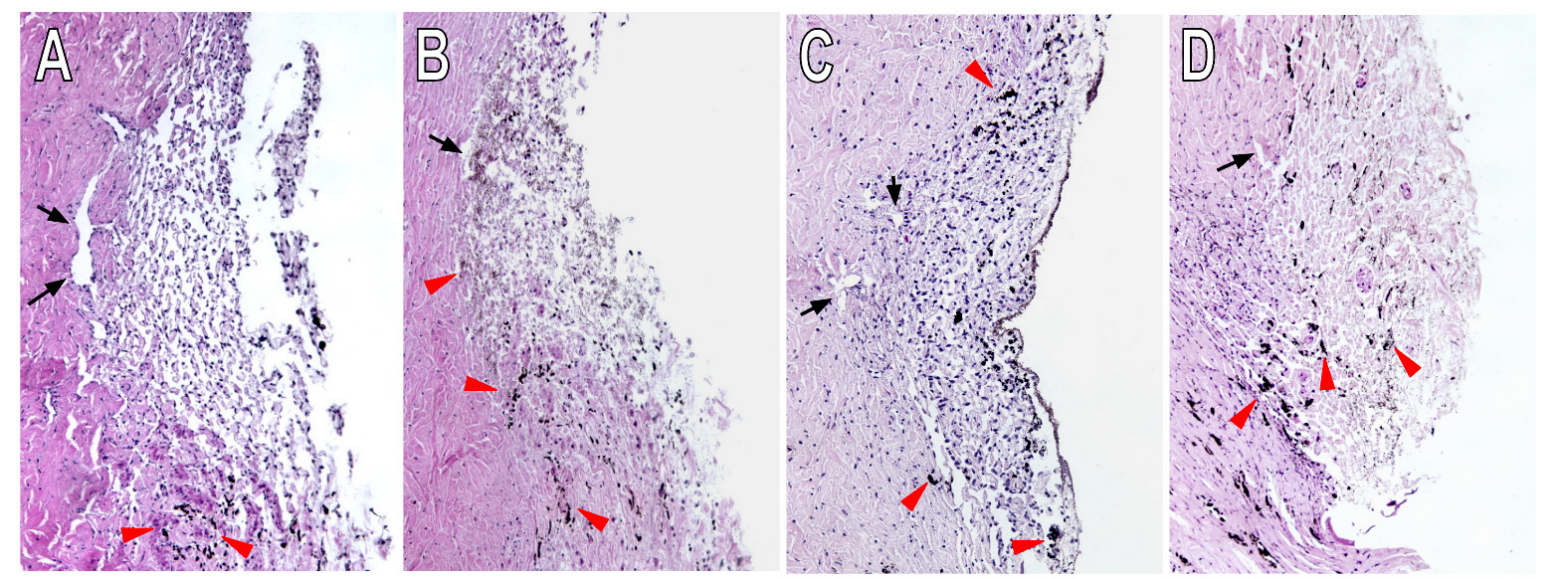
Histology. Normal trabecular meshwork (TM) ( **A**) was a multilayer, strainer-like structure with few pigment deposits (red arrowheads). *Ex vivo* perfusion with pigment granules at 1.67×10 ^7^/ml caused significant TM pigmentation at 48 hours ( **B**), 120 hours ( **C**) and 180 hours ( **D**). No apparent occlusion of the outflow tract was found.

In H&E stained tissue sections, normal TM ( Figure 1A) presented as a sparsely pigmented (red arrowheads), multilayered, porous tissue with Schlemm’s canal-like segments within the aqueous plexus at the outer layer (black arrows). Pigment granules were seen phagocytosed by trabecular meshwork cells, particularly in the uveal TM, at 48, 120, and 180 hours ( Figure 1B, C and D) but were not dense enough to physically obstruct any part of the conventional outflow system.

**Figure 2.**
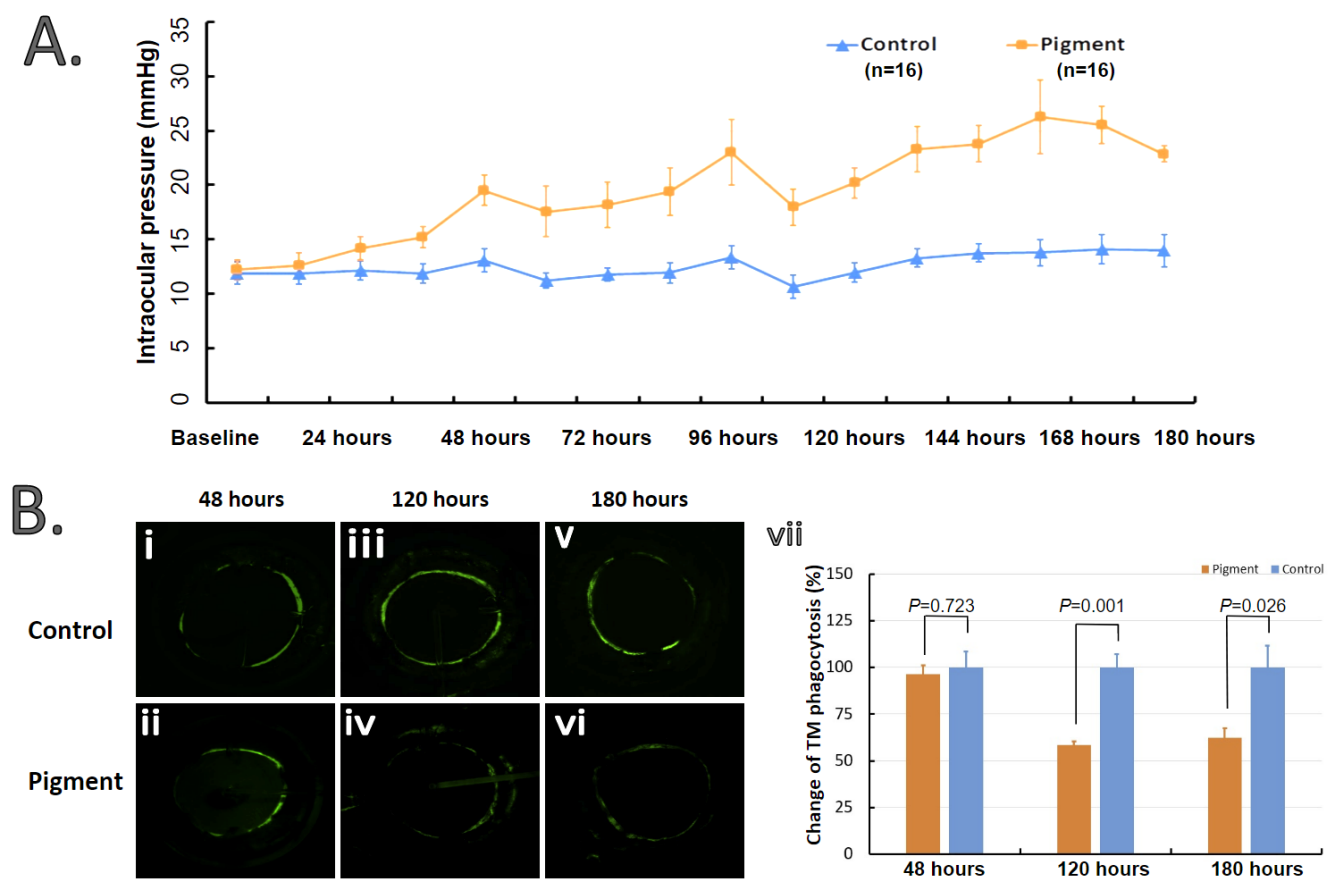
Reduction of intraocular pressure and TM phagocytic activity by pigment dispersion. Baseline IOPs in the pigment group (n=16) and the control (n=16) are comparable (12.2±0.9 mmHg vs. 11.9±0.9 mmHg, *P*=0.82). Pigment caused a significant IOP elevation at 48 hours and onward (all *P*<0.05) while the IOP in the control group showed no significant difference to baseline at any time point (all *P*>0.05) when compared to the baseline ( **A**). TM phagocytosis was visualized *in situ*. The mean fluorescence intensity in the TM region was quantified by NIH ImageJ. TM phagocytosis in the pigment group was comparable to the control at 48 hours ( *P*=0.723), ( **Bi–ii**) but showed sharp decreases at 120 hours ( **Biii–iv**) and 180 hours ( *P*=0.001 and *P*=0.026, respectively) ( **Bv–vi**).

Baseline IOP in Pg was comparable to C (12.2±0.9 mmHg vs. 11.9±0.9 mmHg, *P*=0.82). Pigment dispersion caused a significant IOP elevation at 48, 120, and 180 hours (19.5±1.4 mmHg, 20.2±1.4 mmHg and 22.8±0.8 mmHg, *P*=0.001, *P*<0.001 and *P*=0.002, compared to baseline) while IOPs in C remained steady (13.1±1.1 mmHg, 12.0±0.9 mmHg and 14.0±1.5 mmHg, all *p* values >0.05, compared to baseline) ( Figure 2A).

By inverting the perfusion dishes and washing away the microspheres in the intertrabecular spaces, the TM phagocytosis was visualized and quantified under an upright dissecting fluorescence microscope. Pigment did not cause any change of phagocytosis during early ocular hypertension at 48 hours ( Figure 2Bi-ii, 96.3±5.0% compared to the control, *P*=0.723), but did cause a reduction at the later phases of 120 hours ( Figure 2Biii-iv, 58.3±2.3%, *P*=0.001) and 180 hours ( Figure2Bv-vi, 62.5±5.1%, *P*=0.026). However, the declining phagocytosis did not result in further elevation of IOP at 120 and 180 hours compared to the initial IOP elevation at 48 hours (20.2±1.4 mmHg and 22.8±0.8 mmHg versus 19.5±1.4 mmHg, both *P*>0.05).

**Figure 3.**
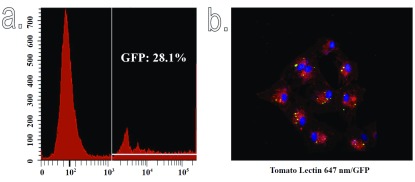
Validation of TM phagocytosis by flow cytometry and confocal microscopy. To further confirm that microspheres were phagocytosed, we digested a normal sample TM tissue into single cell suspension and sent for flow cytometry. The results suggested that 28.1% of the TM cells were actively phagocytic ( **A**). We then seeded these cells into a six well plate to form monolayer. After labeling them with tomato lectin, the confocal imaging showed that clusters of green fluorescent microspheres were located in the intracellular but not in the intercellular space ( **B**).

The microsphere ingestion by TM cells was further assessed by flow cytometry and confocal microscopy. 28.1% of TM cells had phagocytosed microbeads in a normal perfusion eye ( Figure 3A) and the confocal microscopy confirmed them as being located within the cells ( Figure 3B) aided by tomato lectin-stained cell membranes and DAPI-stained nuclei. Confocal imaging showed clusters of green fluorescent microspheres within the intracellular space with no microspheres in the intercellular space. The 3D video also suggested the microspheres were in fact phagocytized and not merely on top of or below them since the microbeads were in the same z plane as the cells ( Supplementary Video 1).

Raw unedited images of Figure 1 They are representative of 17 slides for histology. Click here for additional data file. Copyright: © 2018 Dang Y et al. 2018 Data associated with the article are available under the terms of the Creative Commons Zero "No rights reserved" data waiver (CC0 1.0 Public domain dedication).

Raw unedited images of Figure 2B They are representative of 31 pictures for phagocytosis measurement. Click here for additional data file. Copyright: © 2018 Dang Y et al. 2018 Data associated with the article are available under the terms of the Creative Commons Zero "No rights reserved" data waiver (CC0 1.0 Public domain dedication).

Raw unedited images of Figure 3B Click here for additional data file. Copyright: © 2018 Dang Y et al. 2018 Data associated with the article are available under the terms of the Creative Commons Zero "No rights reserved" data waiver (CC0 1.0 Public domain dedication).

The FACS output file for Figure 3A Click here for additional data file. Copyright: © 2018 Dang Y et al. 2018 Data associated with the article are available under the terms of the Creative Commons Zero "No rights reserved" data waiver (CC0 1.0 Public domain dedication).

The raw IOP and phagocytosis measurements at all time points Click here for additional data file. Copyright: © 2018 Dang Y et al. 2018 Data associated with the article are available under the terms of the Creative Commons Zero "No rights reserved" data waiver (CC0 1.0 Public domain dedication).

## Discussion

Phagocytosis is a defining feature of TM cells ^22^ and plays a central but poorly understood role in the pathogenesis of secondary glaucomas caused by particulate matter such as erythrocytes and ghost cells, inflammatory cells, outer photoreceptor segments, lens and pseudoexfoliation material and pigmentary debris ^8, 23– 25^. These may interfere with various intracellular processes resulting in a gradual deterioration of outflow ^23, 26^. Although TM phagocytosis can remove particles from the aqueous humor ^27^, the direct, short-term effects on outflow regulation remain insufficiently explained ^7^. In this study, we measured IOP and TM phagocytic activity in the presence of pigment granules at different time points and found IOP was significantly elevated as early as 48 hours after exposure to pigment granules. This was contrasted with a phagocytic activity in Pg not different from C before the decrease at 120 and 180 hours. A worsening decline of TM phagocytosis at 120 and 180 hours did not result in a further increase of IOP. This suggests that reduction in phagocytosis is a downstream and secondary effect of actin cytoskeletal reorganization.

Past investigations suggested that the outflow obstruction was, in fact, physical ^12, 28^ but newer studies indicated that ocular hypertension is in part caused indirectly by reorganization of the TM actin cytoskeleton ^29^. This occurs at a concentration far lower (10,000-fold) ^8^ than that used in previous bolus experiments ^12^. Consistent with Zhou *et al.* ^29^, we recently reported that long, thick, and continuous TM actin bundles emerge as early as 24 hours after pigment exposure ^8^ and replicate this observation in the present study. Histological characteristics of pigment dispersion in porcine eyes matched those seen in samples from pigmentary glaucoma patients ^28, 30, 31^ showing that pigment particles were taken up by TM cells.

In summary, the results confirm that the IOP elevation caused by pigment dispersion is not the direct result of physical obstruction of outflow or a chronically overwhelmed phagocytosis. The reduction in phagocytosis considerably lags the evolving hypertension supporting the notion that these cytoskeletal changes occur early on and are separate from the impact of pigment on canonical phagocytosis pathways ^8^.

## Data availability

The data referenced by this article are under copyright with the following copyright statement: Copyright: © 2018 Dang Y et al.

Data associated with the article are available under the terms of the Creative Commons Zero "No rights reserved" data waiver (CC0 1.0 Public domain dedication). 

All the raw data generated or analyzed in this study are included in following datasets.

**Dataset 1.** Raw unedited images of Figure 1. They are representative of 17 slides for histology. 10.5256/f1000research.13797.d192088 ^24^

**Dataset 2.** Raw unedited images of Figure 2B. They are representative of 31 pictures for phagocytosis measurement. 10.5256/f1000research.13797.d192089 ^25^

**Dataset 3.** Raw unedited images of Figure 3B. 10.5256/f1000research.13797.d192090 ^26^

**Dataset 4.** The FACS output file for Figure 3A. 10.5256/f1000research.13797.d192091 ^27^

**Dataset 5.** The raw IOP and phagocytosis measurements at all time points. 10.5256/f1000research.13797.d192092 ^28^

## Supplementary material

**Supplementary Video 1. Visualization of microsphere ingestion by a 3D reconstruction with confocal microscopy**. We took a series of z-stack confocal microscopy images to reconstruct a 3D video, showing that the fluorescent microspheres were neither on the top nor below, but phagocytized by the TM cells.

## References

[1] Tamm ER: The trabecular meshwork outflow pathways: structural and functional aspects. *Exp Eye Res.* 2009;88(4):648–55. 10.1016/j.exer.2009.02.007 19239914

[2] Saccà SC Gandolfi S Bagnis A: The Outflow Pathway: A Tissue With Morphological and Functional Unity. *J Cell Physiol.* 2016;231(9):1876–1893. 10.1002/jcp.25305 26754581

[3] Ethier CR Coloma FM Sit AJ: Two pore types in the inner-wall endothelium of Schlemm’s canal. *Invest Ophthalmol Vis Sci.* 1998;39(11):2041–2048. 9761282

[4] Alvarado JA Betanzos A Franse-Carman L: Endothelia of Schlemm’s canal and trabecular meshwork: distinct molecular, functional, and anatomic features. *Am J Physiol Cell Physiol.* 2004;286(3):C621–C634. 10.1152/ajpcell.00108.2003 14613887

[5] Johnstone MA Grant WG: Pressure-dependent changes in structures of the aqueous outflow system of human and monkey eyes. *Am J Ophthalmol.* 1973;75(3):365–383. 10.1016/0002-9394(73)91145-8 4633234

[6] Braakman ST Daniel Stamer W Overby DR: A fluorescent permeability assay for Schlemm’s canal endothelial cells in response to stretch. *Invest Ophthalmol Vis Sci.* 2014;55(13):5983 Reference Source

[7] Llobet A Gasull X Gual A: Understanding trabecular meshwork physiology: a key to the control of intraocular pressure? *News Physiol Sci.* 2003;18(5):205–9. 10.1152/nips.01443.2003 14500801

[8] Dang Y Waxman S Wang C: A porcine *ex vivo* model of pigmentary glaucoma. *Sci Rep.* 2018;8(1): 5468. 10.1038/s41598-018-23861-x 29615741 PMC5882895

[9] Pattabiraman PP Rao PV: Mechanistic basis of Rho GTPase-induced extracellular matrix synthesis in trabecular meshwork cells. *Am J Physiol Cell Physiol.* 2010;298(3):C749–63. 10.1152/ajpcell.00317.2009 19940066 PMC2838580

[10] Tanihara H Inoue T Yamamoto T: Intra-ocular pressure-lowering effects of a Rho kinase inhibitor, ripasudil (K-115), over 24 hours in primary open-angle glaucoma and ocular hypertension: a randomized, open-label, crossover study. *Acta Ophthalmol.* 2015;93(4):e254–60. 10.1111/aos.12599 25487877

[11] Tanihara H Inoue T Yamamoto T: Phase 1 clinical trials of a selective Rho kinase inhibitor, K-115. *JAMA Ophthalmol.* 2013;131(10):1288–95. 10.1001/jamaophthalmol.2013.323 23787820

[12] Epstein DL Freddo TF Anderson PJ: Experimental obstruction to aqueous outflow by pigment particles in living monkeys. *Invest Ophthalmol Vis Sci.* 1986;27(3):387–95. 3949467

[13] Campbell DG Simmons RJ Grant WM: Ghost cells as a cause of glaucoma. *Am J Ophthalmol.* 1976;81(4):441–50. 10.1016/0002-9394(76)90299-3 1266922

[14] Moorthy RS Mermoud A Baerveldt G: Glaucoma associated with uveitis. *Surv Ophthalmol.* 1997;41(5):361–94. 10.1016/S0039-6257(97)00006-4 9163835

[15] Callender D Jay JL Barrie T: Schwartz-Matsuo syndrome: atypical presentation as acute open angle glaucoma. *Br J Ophthalmol.* 1997;81(7):609–10. 10.1136/bjo.81.7.608b 9290381 PMC1722260

[16] Lee RK: The molecular pathophysiology of pseudoexfoliation glaucoma. *Curr Opin Ophthalmol.* 2008;19(2):95–101. 10.1097/ICU.0b013e3282f49cda 18301281

[17] Ritch R Schlötzer-Schrehardt U Konstas AG: Why is glaucoma associated with exfoliation syndrome? *Prog Retin Eye Res.* 2003;22(3):253–75. 10.1016/S1350-9462(02)00014-9 12852486

[18] Loewen RT Roy P Park DB: A Porcine Anterior Segment Perfusion and Transduction Model With Direct Visualization of the Trabecular Meshwork. *Invest Ophthalmol Vis Sci.* 2016;57(3):1338–44. 10.1167/iovs.15-18125 27002293 PMC4811178

[19] Dang Y Waxman S Wang C: Freeze-thaw decellularization of the trabecular meshwork in an *ex vivo* eye perfusion model. *Peer J Preprints.* 2017;5: e2736v1. 10.7287/peerj.preprints.2736v1 PMC5560227 28828244

[20] Loewen RT Brown EN Scott G: Quantification of Focal Outflow Enhancement Using Differential Canalograms. *Invest Ophthalmol Vis Sci.* 2016;57(6):2831–8. 10.1167/iovs.16-19541 27227352 PMC5113980

[21] Dang Y Waxman S Wang C: Rapid learning curve assessment in an *ex vivo* training system for microincisional glaucoma surgery. *PeerJ Preprints.* 2017;5:e2745v1 10.7287/peerj.preprints.2745v1 PMC5431621 28487512

[22] Buller C Johnson DH Tschumper RC: Human trabecular meshwork phagocytosis. Observations in an organ culture system. *Invest Ophthalmol Vis Sci.* 1990;31(10):2156–63. 2211012

[23] Liton PB Lin Y Gonzalez P: Potential role of lysosomal dysfunction in the pathogenesis of primary open angle glaucoma. *Autophagy.* 2009;5(1):122–4. 10.4161/auto.5.1.7304 19001861 PMC2745819

[24] Zhang X Ognibene CM Clark AF: Dexamethasone inhibition of trabecular meshwork cell phagocytosis and its modulation by glucocorticoid receptor beta. *Exp Eye Res.* 2007;84(2):275–84. 10.1016/j.exer.2006.09.022 17126833 PMC1796996

[25] Sivaraman KR Patel CG Vajaranant TS: Secondary pigmentary glaucoma in patients with underlying primary pigment dispersion syndrome. *Clin Ophthalmol.* 2013;7:561–566. 10.2147/OPTH.S42456 23569351 PMC3615837

[26] Saccà SC Gandolfi S Bagnis A: From DNA damage to functional changes of the trabecular meshwork in aging and glaucoma. *Ageing Res Rev.* 2016;29:26–41. 10.1016/j.arr.2016.05.012 27242026

[27] Abu-Hassan DW Acott TS Kelley MJ: The Trabecular Meshwork: A Basic Review of Form and Function. *J Ocul Biol.* 2014;2(1): pii: http://fulltextarticles.avensonline.org/JOCB-2334-2838-02-0017.html. 10.13188/2334-2838.1000017 25356439 PMC4209746

[28] Alvarado JA Murphy CG: Outflow obstruction in pigmentary and primary open angle glaucoma. *Arch Ophthalmol.* 1992;110(12):1769–78. 10.1001/archopht.1992.01080240109042 1463421

[29] Zhou L Li Y Yue BY: Alteration of cytoskeletal structure, integrin distribution, and migratory activity by phagocytic challenge in cells from an ocular tissue--The trabecular meshwork. *In Vitro Cell Dev Biol Anim.* 1999;35(3):144–149. 10.1007/s11626-999-0016-6 10476910

[30] Gottanka J Johnson DH Grehn F: Histologic findings in pigment dispersion syndrome and pigmentary glaucoma. *J Glaucoma.* 2006;15(2):142–51. 10.1097/00061198-200604000-00011 16633228

[31] Kupfer C Kuwabara T Kaiser-Kupfer M: The histopathology of pigmentary dispersion syndrome with glaucoma. *Am J Ophthalmol.* 1975;80(5):857–62. 10.1016/0002-9394(75)90283-4 1190279

