# Supplementary figures and images for: Intraocular pressure elevation precedes a phagocytosis decline in a model of pigmentary glaucoma
Source: F1000Res. 2018 Apr 9;7:174. Originally published 2018 Feb 12. [Version 2] doi: 10.12688/f1000research.13797.2 (PMC5915754; doi:10.12688/f1000research.13797.2)

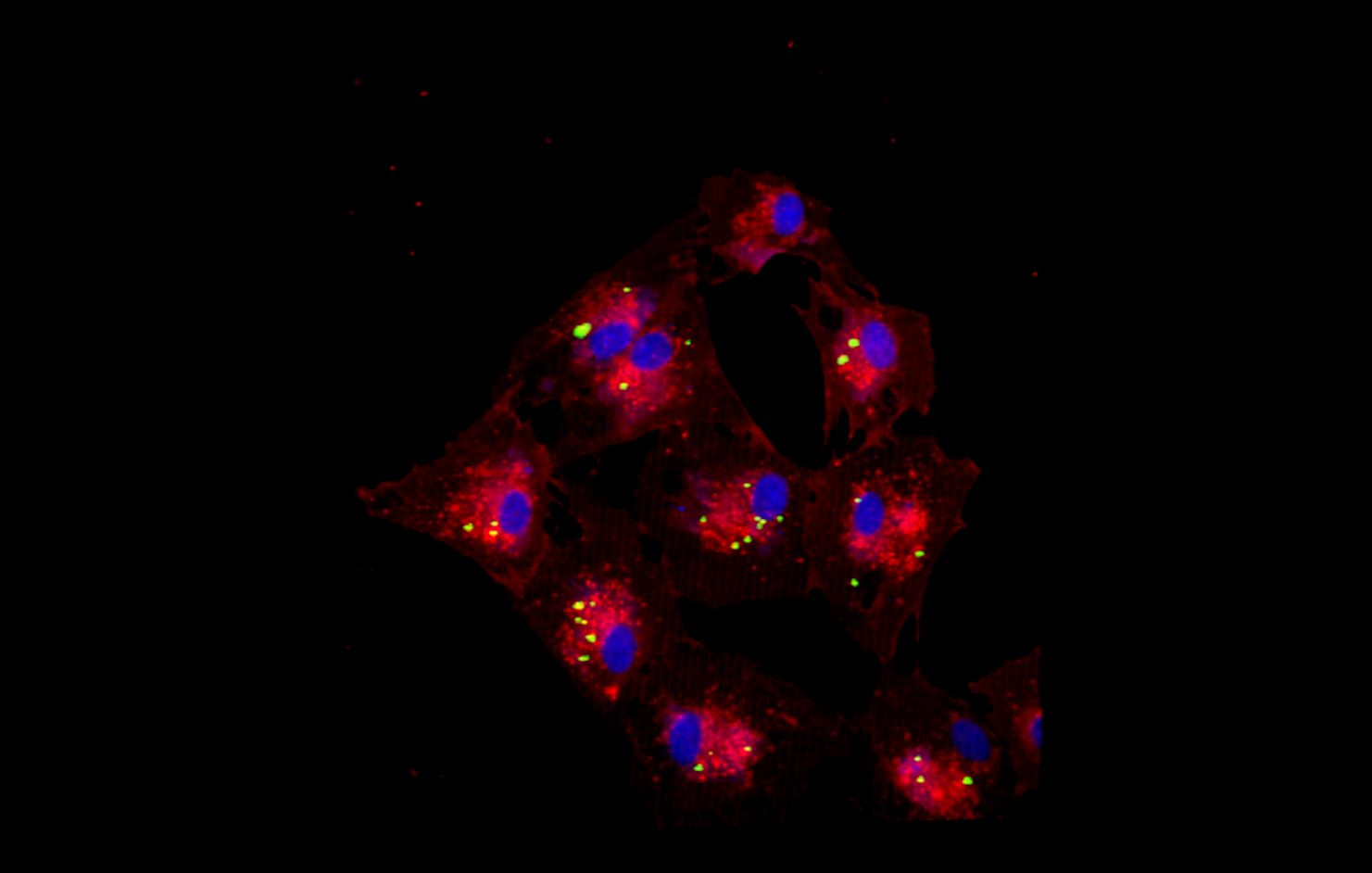

Supplement: Raw unedited images of Figure 3B [file f1000research-7-15759-s0002.tgz › 79b4963d-0524-49de-aca8-7d99bbb73055_Supplementary_Dataset_3._Raw_unedited_images_of_Figure_3B..tif]
