# Supplementary material for: Intraocular pressure elevation precedes a phagocytosis decline in a model of pigmentary glaucoma
Source: F1000Res. 2018 Apr 9;7:174. Originally published 2018 Feb 12. [Version 2] doi: 10.12688/f1000research.13797.2 (PMC5915754; doi:10.12688/f1000research.13797.2)
Supplement: The FACS output file for Figure 3A [file f1000research-7-15759-s0003.tgz › a5e65beb-96b5-4f63-b398-1de7627c1cfc_Supplementary_Dataset_4._The_FACS_output_file_for_Figure_3A..pdf]

FACSDiva Version 6.1.3

Specimen Na...	111517	
Tube Name:	TM Sample	
**Population**	**#Events**	**%Parent**
GFP-1	6,538	28.1

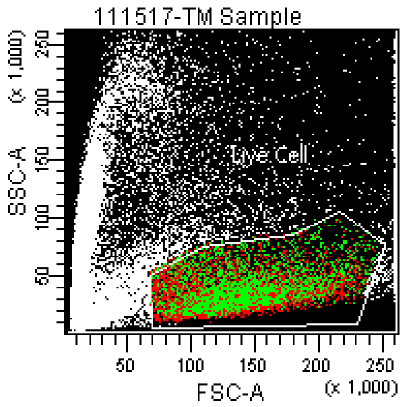

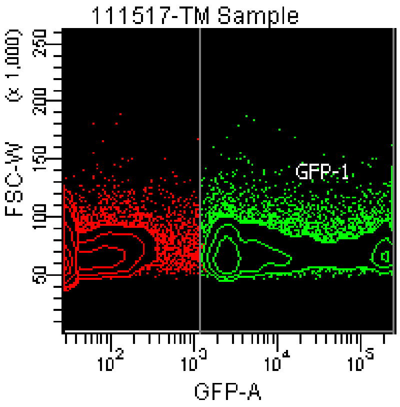

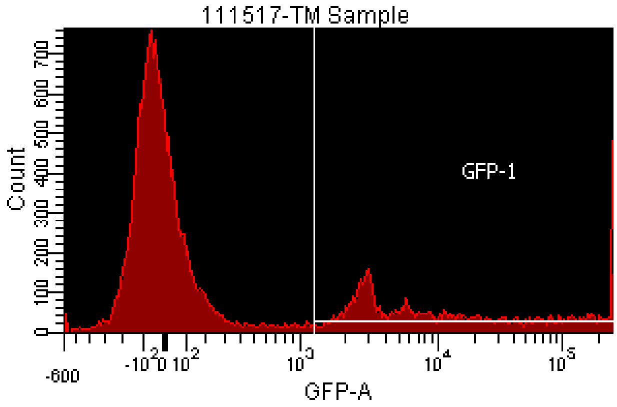

Tube: TM Sample

Population	#Events	%Parent	%Total
All Events	200,000	####	100.0
Live Cell	23,306	11.7	11.7
GFP-1	6,538	28.1	3.3
