# Supplementary material for: Intraocular pressure elevation precedes a phagocytosis decline in a model of pigmentary glaucoma
Source: F1000Res. 2018 Apr 9;7:174. Originally published 2018 Feb 12. [Version 2] doi: 10.12688/f1000research.13797.2 (PMC5915754; doi:10.12688/f1000research.13797.2)
Supplement: Peer review history

# Peer review: Referee response for version 2

**DOI:** 10.5256/f1000research.15759.r33294

**Published:** 2018-04-23

**Authors:** Don S. Minckler

**Affiliations:** 1 Gavin Herbert Eye Institute, University of California, Irvine, CA, USA

In my view, this well written report adds clarification to an old problem of explaining the pathogenesis of glaucoma secondary to particulate material in the aqueous.  The authors’ conclusions should direct additional attention to studying the biomechanics of meshwork functioning especially phagocytosis by the lining endothelial cells.  The experimental methods are complex but make sense considering the challenges of the physiology being investigated.

 I have no major criticisms and suggest the paper be indexed as is.

I have read this submission. I believe that I have an appropriate level of expertise to confirm that it is of an acceptable scientific standard.

# Peer review: Referee response for version 2

**DOI:** 10.5256/f1000research.15759.r32895

**Published:** 2018-04-12

**Authors:** Sergio C Saccà

**Affiliations:** 1 Department of Neuroscience and Sense Organs, RCCS San Martino University Hospital, Genoa, Italy

I read with interest the new version of the article by Yalong Dang et al. And I'm glad to find out that the authors' ideas match up with mine. I believe that in pigmentary glaucoma there is a defect in the autophagy of the cells of Trabecolato and therefore this version is more in keeping with modern biology. I believe it can now be published.

I have read this submission. I believe that I have an appropriate level of expertise to confirm that it is of an acceptable scientific standard.

# Peer review: Referee response for version 1

**DOI:** 10.5256/f1000research.14999.r32109

**Published:** 2018-03-26

**Authors:** Sergio C Saccà

**Affiliations:** 1 Department of Neuroscience and Sense Organs, RCCS San Martino University Hospital, Genoa, Italy

I read the article titled: Intraocular pressure elevation precedes a phagocytosis decline in a model of pigmentary glaucoma by Dang *et al.* The title of the article is very stimulating but the content has several things that should be corrected. The authors state that in the conventional way of outflow the aqueous passes into Schlemm's canal by paracytosis or giant vacuoles. This is a partial view that does not correspond to the truth. I invite the authors to read Saccà et al. The Outflow Pathway: A Tissue With Morphological and Functional Unity ^1^. Then the effects of the inhibitors of the Rho kinase inhibitor may have the ability to modify the metabolism of endothelial cells in the node in which the authors think it is open to question. These drugs have the ability to block TM motility by exposing more cells to aqueous humor. This is the reason why we initially witness a decline in IOP then this worsens because the exposed cells are blocked and are more exposed to apoptosis (to read Saccà *et al.* from DNA damage to functional changes of the trabecular meshwork in aging and glaucoma ^2^). Also the hypothesis formulated by the authors to explain the pigmentary glaucoma is partial in the Trabecolato the so-called pores have a very low meaning and above all the pigment does not obstruct anything. We do not work like sinks -  I am amazed that today we can still believe that TM is a porous tissue, and above all, how it is possible to think that outflow is a passive phenomenon. In pigmentary glaucoma there are 2 fundamental pathogenic moments. The first concerns the back sheet of the iris, where the pigment comes from (a defect that involves the loss of the pigment itself) the second point concerns autophagy which is not able to ensure adequate cell homeostasis. So these cells first enter suffering and then die and hence glaucoma is why the cells do not work, not because it obstructs something. There are cells, not tubes. The cytoskeleton in all this has little to do with it: pigment granules are only a metabolic burden that in the case of pigment dispersal syndrome is absorbed by a functioning autophagy, while in pigment glaucoma, autophagy does not work. Finally, I remember that the changes in the cytoskeleton occur in all types of glaucoma. I do not believe that this article can be indexed in this form and needs a remake of both the introduction and the discussion and the results should be reviewed in the light of a more modern interpretation of the physio-pathogenical events.

I have read this submission. I believe that I have an appropriate level of expertise to state that I do not consider it to be of an acceptable scientific standard, for reasons outlined above.

## References

[1] SaccàSCGandolfiSBagnisAManniGDamonteGTraversoCEIzzottiA: The Outflow Pathway: A Tissue With Morphological and Functional Unity. *J Cell Physiol*.2016;231(9) : 10.1002/jcp.25305 1876-93 10.1002/jcp.25305 26754581

[2] SaccàSCGandolfiSBagnisAManniGDamonteGTraversoCEIzzottiA: From DNA damage to functional changes of the trabecular meshwork in aging and glaucoma. *Ageing Res Rev*.2016;29: 10.1016/j.arr.2016.05.012 26-41 10.1016/j.arr.2016.05.012 27242026

# Response

**Published:** 2018-03-31

**Authors:** Nils Loewen

**Affiliations:** University of Pittsburgh Medical Center, USA

**Reviewer 1:**

 I read the article titled: Intraocular pressure elevation precedes a phagocytosis decline in a model of pigmentary glaucoma by Dang et al. The title of the article is very stimulating but the content has several things that should be corrected.

**Authors:** We thank the reviewer, Professor Saccà, for having taken the time to carefully review our manuscript. We hope that we are able to address all of them with extensive manuscript changes as requested.

**Reviewer 1:**

**1)**The authors state that in the conventional way of outflow the aqueous passes into Schlemm's canal by paracytosis or giant vacuoles. This is a partial view that does not correspond to the truth. I invite the authors to read Saccà et al. The Outflow Pathway: A Tissue With Morphological and Functional Unity 1.

**Authors: **Thank you for bringing this review article to our attention. We are pleased to use this publication and your other article as additional references. We have modified the Introduction to include  more comprehensive details about conventional outflow better aligned with Saccà et al. We would like to change the less common phrase “paracytosis” with “paracellular passage through pores” to distinguish it from the active process of pathogen intrusion through epithelia e.g. by haemophilus. 1 Overall, it appears our manuscript matches Saccà et al well who state that the main mechanisms of aqueous outflow are “a paracellular route [...] and a transcellular pathway”. We have added to the Introduction the description of a transcellular pathway through intracellular pores 2 with inducible pore density that depends on outflow demand 3.

In the reworded Introduction we now state: “The aqueous passes from the anterior chamber into Schlemm's canal (SC) by entering first the uveal TM (UTM), the corneoscleral TM (CTM) and finally the juxtacanalicular TM (JCT) 4. The JCT contains proteoglycans and hyaluronans and presents the aqueous humor with an increasingly tighter fluid passageway towards the SC in a process referred to as funneling 5. The aqueous eventually passes the inner wall of SC endothelium mainly by two different mechanisms, a paracellular route in between endothelial cells and a transcellular route 6  consisting of intracellular pores and giant vacuoles that are time and pressure dependent 7–9. Failure of the TM and the inner wall of SC endothelial cells to maintain homeostasis and a normal cytoskeleton and can cause ocular hypertension 10. For instance, pigment dispersion 11 and corticosteroids can increase and contract actin stress fibers and result in an elevation of the intraocular pressure (IOP) 11,12. Conversely, relaxing the cytoskeleton with Rho kinase inhibitors can reverse these effects 13,14. Phagocytosis of debris is another function of TM cells 8. A chronic phagocytosis demand in the form of pigment 15, erythrocyte-derived ghost cells 16, inflammatory cells 17, photoreceptor outer segments 18, lens and pseudoexfoliation material 19,20 can all lead to secondary glaucomas even though the amount of material itself is unlikely to cause a physical outflow obstruction.  Although these glaucomas make for a sizable fraction of open angle glaucomas, it was difficult to study the cellular mechanism that leads to an IOP elevation.”

**2) **Then the effects of the inhibitors of the Rho kinase inhibitor may have the ability to modify the metabolism of endothelial cells in the node in which the authors think it is open to question. These drugs have the ability to block TM motility by exposing more cells to aqueous humor. This is the reason why we initially witness a decline in IOP then this worsens because the exposed cells are blocked and are more exposed to apoptosis (to read Saccà et al. from DNA damage to functional changes of the trabecular meshwork in aging and glaucoma 2).

**Authors:** We completely agree with the Reviewer. We would like to point out that - consistent with Sacca et al - we wrote that “we hypothesized that ocular hypertension is the result of a reorganization of the actin cytoskeleton and occurs before phagocytosis declines.” We did not intend to describe outflow through the TM as sink-like. We hope the expanded Introduction makes it more obvious that this is a paracellular and transcellular process.

 In addition, we specifically stated before that “material itself is unlikely to cause a physical outflow obstruction” (Introduction), that granules “were not dense enough to physically obstruct any part of the conventional outflow system” (Results) and discuss that this had already been shown before: “Pigment treatment has previously been shown to cause ocular hypertension in part by reorganizing the TM actin cytoskeleton and not by physical obstruction of the outflow tract 15,21.” (Discussion).

 We now clarify in the Discussion that “Past investigations suggested that the outflow obstruction was, in fact, physical 15,22 but newer studies indicated that ocular hypertension is in part caused indirectly by reorganization of the TM actin cytoskeleton 23. Consistent with Zhou et al. 23, we recently reported that long, thick, and continuous TM actin bundles emerge as early as 24 hours after pigment exposure 11 and replicate this observation in the present study.”

**3)** Also the hypothesis formulated by the authors to explain the pigmentary glaucoma is partial in the Trabecolato the so-called pores have a very low meaning and above all the pigment does not obstruct anything. We do not work like sinks -  I am amazed that today we can still believe that TM is a porous tissue, and above all, how it is possible to think that outflow is a passive phenomenon.

**Authors: **We did not state anything about an outflow obstruction or a location in our hypothesis. We wrote, “In the current study, we hypothesized that ocular hypertension is the result of a reorganization of the actin cytoskeleton and occurs before phagocytosis declines.” As we detail in our Response to 3), we never wrote that the TM functions as a sink. On the contrary, we elaborate multiple times that there is no physical obstruction as detailed in our Response to 2). Please see our response above where we hope to clarify this point in the rewritten manuscript.

**4)** In pigmentary glaucoma there are 2 fundamental pathogenic moments. The first concerns the back sheet of the iris, where the pigment comes from (a defect that involves the loss of the pigment itself) the second point concerns autophagy which is not able to ensure adequate cell homeostasis. So these cells first enter suffering and then die and hence glaucoma is why the cells do not work, not because it obstructs something. There are cells, not tubes.

**Authors:** We completely agree as detailed in 1)-3) and have expanded our manuscript to make this more obvious.

**5)** The cytoskeleton in all this has little to do with it: pigment granules are only a metabolic burden that in the case of pigment dispersal syndrome is absorbed by a functioning autophagy, while in pigment glaucoma, autophagy does not work. Finally, I remember that the changes in the cytoskeleton occur in all types of glaucoma. I do not believe that this article can be indexed in this form and needs a remake of both the introduction and the discussion and the results should be reviewed in the light of a more modern interpretation of the physio-pathogenical events.

**Authors:** We are unsure why the Reviewer states that cytoskeletal changes have nothing to do with an IOP elevation after a phagocytosis challenge. This is rather well established: Zhou et al found that 4 h after phagocytosis, the cytoskeletal structure in trabecular meshwork cells was disrupted 23. As we detail in the Introduction “pigment dispersion 11 and corticosteroids can increase and contract actin stress fibers and result in an elevation of the intraocular pressure (IOP) 11,12. Conversely, relaxing the cytoskeleton with Rho kinase inhibitors can reverse these effects 13,14.”

 The new finding described in this manuscript is that the IOP rises when the cytoskeleton changes occur as a result form pigment exposure but before phagocytosis starts to fail. This has not been observed before because an appropriate ex vivo anterior segment model of pigment dispersion did not exist.

 Our recent studies 11,24,25 show that ex vivo perfused pig eyes experience reduced outflow in response to continuous exposure to pigment at a concentration far lower (10,000-fold) than that used in previous bolus experiments 26 and that severe cytoskeletal changes are associated with this. Conversely, we show that rho-kinase inhibitors normalize the cytoskeleton and the phagocytosis 24.  TM regulates aqueous outflow by changing its cytoskeleton, stiffness, cell adhesion, migration, contraction, and phagocytosis 10,27, in which ROCK signaling plays a central role 28,29. There is a close interaction between actin cytoskeleton reorganization, cell or extracellular matrix stiffness and aqueous outflow facility 30,31. Only a small proportion of stress fiber formation was found in the normal TM culture while pigment exposure increased it by 2.06-fold 11.

 Lastly, we caution not to confuse TM cell phagocytosis of pigmented debris with autophagy. To address the Reviewer’s concern we have added to the Discussion his own reference 32 and state that “An increased pigmentary debris may interfere with many intracellular functions, including important autophagy functions that can cause a gradual deterioration of TM cell function 32,33.”  

**References used by Reviewer 1**

 1. Saccà SC, Gandolfi S, Bagnis A, Manni G, Damonte G, Traverso CE, Izzotti A: The Outflow Pathway: A Tissue With Morphological and Functional Unity.J Cell Physiol. 2016; 231 (9): 1876-93 PubMed Abstract | Publisher Full Text

 2. Saccà SC, Gandolfi S, Bagnis A, Manni G, Damonte G, Traverso CE, Izzotti A: From DNA damage to functional changes of the trabecular meshwork in aging and glaucoma.Ageing Res Rev. 2016; 29: 26-41 PubMed Abstract | Publisher Full Text

**References used by the Authors in this Reply**

 1. van Schilfgaarde M, van Alphen L, Eijk P, Everts V, Dankert J. Paracytosis of Haemophilus influenzae through cell layers of NCI-H292 lung epithelial cells. Infect Immun. 1995 Dec;63(12):4729–4737. PMCID: PMC173678

 2. Johnson M, Erickson K. Mechanisms and routes of aqueous humor drainage. Principles and Practice of Ophthalmology. Saunders Philadelphia; 2000;4:2577–2595.

 3. Braakman ST, Pedrigi RM, Read AT, Smith JAE, Stamer WD, Ethier CR, Overby DR. Biomechanical strain as a trigger for pore formation in Schlemm’s canal endothelial cells. Exp Eye Res. 2014 Oct;127:224–235. PMCID: PMC4175173

 4. Saccà SC, Gandolfi S, Bagnis A, Manni G, Damonte G, Traverso CE, Izzotti A. The Outflow Pathway: A Tissue With Morphological and Functional Unity. J Cell Physiol. 2016 Sep;231(9):1876–1893. PMID: 26754581

 5. Ethier CR, Coloma FM, Sit AJ, Johnson M. Two pore types in the inner-wall endothelium of Schlemm’s canal. Invest Ophthalmol Vis Sci. 1998;39:2041–2048.

 6. Alvarado JA, Betanzos A, Franse-Carman L, Chen J, González-Mariscal L. Endothelia of Schlemm’s canal and trabecular meshwork: distinct molecular, functional, and anatomic features. Am J Physiol Cell Physiol. 2004 Mar;286(3):C621–34. PMID: 14613887

 7. Johnstone MA, Grant WM. Pressure-dependent changes in structures of the aqueous outflow system of human and monkey eyes. Am J Ophthalmol. 1973 Mar;75(3):365–383. PMID: 4633234

 8. Tamm ER. The trabecular meshwork outflow pathways: structural and functional aspects. Exp Eye Res. 2009 Apr;88(4):648–655. PMID: 19239914

 9. Braakman ST, Daniel Stamer W, Overby DR. A fluorescent permeability assay for Schlemm’s canal endothelial cells in response to stretch. Invest Ophthalmol Vis Sci. The Association for Research in Vision and Ophthalmology; 2014 Apr 30;55(13):5983–5983.

 10. Llobet A, Gasull X, Gual A. Understanding trabecular meshwork physiology: a key to the control of intraocular pressure? News Physiol Sci. 2003 Oct;18:205–209. PMID: 14500801

 11. Dang Y, Waxman S, Wang C, Loewen RT, Sun M, Loewen N. A porcine ex vivo model of pigmentary glaucoma. Sci Rep [Internet]. 2018 Mar 26;8. Available from: http://dx.doi.org/10.1038/s41598-018-23861-x

 12. Pattabiraman PP, Rao PV. Mechanistic basis of Rho GTPase-induced extracellular matrix synthesis in trabecular meshwork cells. Am J Physiol Cell Physiol. 2010 Mar;298(3):C749–63. PMCID: PMC2838580

 13. Tanihara H, Inoue T, Yamamoto T, Kuwayama Y, Abe H, Suganami H, Araie M, K-115 Clinical Study Group. Intra-ocular pressure-lowering effects of a Rho kinase inhibitor, ripasudil (K-115), over 24 hours in primary open-angle glaucoma and ocular hypertension: a randomized, open-label, crossover study. Acta Ophthalmol. 2015 Jun;93(4):e254–60. PMID: 25487877

 14. Tanihara H, Inoue T, Yamamoto T, Kuwayama Y, Abe H, Araie M, K-115 Clinical Study Group. Phase 1 clinical trials of a selective Rho kinase inhibitor, K-115. JAMA Ophthalmol. 2013 Oct;131(10):1288–1295. PMID: 23787820

 15. Epstein DL, Freddo TF, Anderson PJ, Patterson MM, Bassett-Chu S. Experimental obstruction to aqueous outflow by pigment particles in living monkeys. Invest Ophthalmol Vis Sci. 1986 Mar;27(3):387–395. PMID: 3949467

 16. Campbell DG, Simmons RJ, Grant WM. Ghost cells as a cause of glaucoma. Am J Ophthalmol. 1976 Apr;81(4):441–450. PMID: 1266922

 17. Moorthy RS, Mermoud A, Baerveldt G, Minckler DS, Lee PP, Rao NA. Glaucoma associated with uveitis. Surv Ophthalmol. 1997 Mar;41(5):361–394. PMID: 9163835

 18. Callender D, Jay JL, Barrie T. Schwartz–Matsuo syndrome: atypical presentation as acute open angle glaucoma. Br J Ophthalmol. BMJ Publishing Group Ltd; 1997 Jul 1;81(7):608–608.

 19. Lee RK. The molecular pathophysiology of pseudoexfoliation glaucoma. Curr Opin Ophthalmol. 2008 Mar;19(2):95–101. PMID: 18301281

 20. Ritch R, Schlötzer-Schrehardt U, Konstas AGP. Why is glaucoma associated with exfoliation syndrome? Prog Retin Eye Res. 2003 May;22(3):253–275. PMID: 12852486

 21. Gottanka J, Johnson DH, Grehn F, Lütjen-Drecoll E. Histologic findings in pigment dispersion syndrome and pigmentary glaucoma. J Glaucoma. 2006 Apr;15(2):142–151. PMID: 16633228

 22. Alvarado JA, Murphy CG. Outflow obstruction in pigmentary and primary open angle glaucoma. Arch Ophthalmol. 1992 Dec;110(12):1769–1778. PMID: 1463421

 23. Zhou L, Li Y, Beatrice Y J. Alteration of cytoskeletal structure, integrin distribution, and migratory activity by phagocytic challenge in cells from an ocular tissue—The trabecular meshwork. In Vitro CellDevBiol-Animal. Springer-Verlag; 1999 Mar 1;35(3):144–149.

 24. Dang Y, Wang C, Shah P, Waxman S, Loewen RT, Loewen NA. Ocular Hypotension, Actin Stress Fiber Disruption and Phagocytosis Increase by RKI-1447, a Rho-Kinase Inhibitor [Internet]. 2018 [cited 2018 Feb 26]. Available from: http://dx.doi.org/10.20944/preprints201802.0026.v1

 25. Wang C, Dang Y, Loewen R, Waxman S, Shah P, Xia X, Loewen NA. Impact of Pigment Dispersion on Trabecular Meshwork Cells. ResearchGate preprint [Internet]. 2018 Jan 25 [cited 2018 Jan 25]; Available from: https://www.researchgate.net/publication/322627138_Impact_of_Pigment_Dispersion_on_Trabecular_Meshwork_Cells

 26. Epstein DL, Freddo TF, Anderson PJ, Patterson MM, Bassett-Chu S. Experimental obstruction to aqueous outflow by pigment particles in living monkeys. Invest Ophthalmol Vis Sci. 1986 Mar;27(3):387–395. PMID: 3949467

 27. Abu-Hassan DW, Acott TS, Kelley MJ. The Trabecular Meshwork: A Basic Review of Form and Function. J Ocul Biol Dis Infor [Internet]. 2014 May;2(1). Available from: https://www.ncbi.nlm.nih.gov/pubmed/25356439 PMCID: PMC4209746

 28. Nakajima E, Nakajima T, Minagawa Y, Shearer TR, Azuma M. Contribution of ROCK in contraction of trabecular meshwork: proposed mechanism for regulating aqueous outflow in monkey and human eyes. J Pharm Sci. 2005 Apr;94(4):701–708. PMID: 15682386

 29. Wang J, Liu X, Zhong Y. Rho/Rho-associated kinase pathway in glaucoma (Review). Int J Oncol. 2013 Nov;43(5):1357–1367. PMID: 24042317

 30. Doornaert B, Leblond V, Planus E, Galiacy S, Laurent VM, Gras G, Isabey D, Lafuma C. Time course of actin cytoskeleton stiffness and matrix adhesion molecules in human bronchial epithelial cell cultures. Exp Cell Res. 2003 Jul 15;287(2):199–208. PMID: 12837276

 31. Wang K, Read AT, Sulchek T, Ethier CR. Trabecular meshwork stiffness in glaucoma. Exp Eye Res. 2017 May 1;158:3–12.

 32. Saccà SC, Gandolfi S, Bagnis A, Manni G, Damonte G, Traverso CE, Izzotti A. From DNA damage to functional changes of the trabecular meshwork in aging and glaucoma. Ageing Res Rev. 2016 Aug;29:26–41. PMID: 27242026

 33. Liton PB, Lin Y, Gonzalez P, Epstein DL. Potential role of lysosomal dysfunction in the pathogenesis of primary open angle glaucoma. Autophagy. 2009 Jan;5(1):122–124. PMCID: PMC2745819
